# Public Health Impact of FDA’s Request for Additional Safety Data on Cytisine for Tobacco Cessation

**DOI:** 10.1001/jamahealthforum.2024.2647

**Published:** 2024-08-23

**Authors:** Krishna P. Reddy, A. David Paltiel, Kenneth A. Freedberg, Nancy A. Rigotti

**Affiliations:** 1 Medical Practice Evaluation Center, Mongan Institute, Massachusetts General Hospital, Boston; 2 Tobacco Research and Treatment Center, Mongan Institute, Massachusetts General Hospital, Boston; 3 Division of Pulmonary and Critical Care Medicine, Massachusetts General Hospital, Boston; 4 Harvard Medical School, Boston, Massachusetts; 5 Public Health Modeling Unit, Yale School of Public Health, New Haven, Connecticut; 6 Division of General Internal Medicine, Massachusetts General Hospital, Boston; 7 Division of Infectious Diseases, Massachusetts General Hospital, Boston; 8 Department of Health Policy and Management, Harvard T.H. Chan School of Public Health, Boston, Massachusetts

## Abstract

**Importance:**

No new tobacco cessation medication has been licensed in the US since 2006. Cytisine, a plant-based partial agonist of nicotinic acetylcholine receptors, has demonstrated safety and efficacy in several randomized clinical trials and is currently available in many countries. However, the drug is not commercially available in the US. A New Drug Application to license cytisine as a smoking cessation medication in the US is being prepared for review by the US Food and Drug Administration, whose request for additional safety data will delay submission of the application by approximately 1 year.

**Objective:**

To project the potential public health impact of cytisine, and delays in its availability, as a smoking cessation aid in the US.

**Design, Setting, and Participants:**

This mathematical model estimated life expectancy gains from smoking cessation for people aged 18 to 99 years in the US, reflecting the civilian, noninstitutionalized population. The model also accounted for cytisine uptake and effectiveness, as well as potential relapse among people who stop smoking.

**Exposure:**

Cytisine availability as a tobacco cessation treatment immediately or after 1 year.

**Main Outcomes and Measures:**

The main outcomes were the number of adults able to stop smoking and sustain long-term abstinence and aggregate life-years gained.

**Results:**

The base case includes an estimated 29.4 million US civilian noninstitutionalized adults who smoke cigarettes (age distribution, 18-24 years: 5.5%; 25-44 years: 37.3%; 45-64 years: 41.8%; ≥65 years: 15.5%). With a conservative assumption that 3.8% of these individuals would use cytisine in the first year of availability, immediate cytisine availability could lead 71 000 more people to quit smoking over 1 year and maintain long-term abstinence. This would produce more than 500 000 additional life-years compared to the status quo in which cytisine is unavailable and fewer people stop smoking. Each additional year of delay in the availability of cytisine might reduce population-level life expectancy by 10 000 years. The model results were most sensitive to changes in cytisine uptake and effectiveness.

**Conclusions and Relevance:**

Smoking cessation generates large gains in life expectancy. This mathematical model demonstrated that immediate cytisine availability, even if used successfully by only a small fraction of people who smoke, could produce major public health benefits. Given the need for new tobacco cessation pharmacotherapy options, the magnitude of cytisine’s potential public health benefits, and the morbidity and mortality associated with delay in its availability, a timely review of cytisine for approval in the US is warranted.

## Key Points

**Question:**

What is the potential public health impact of cytisine, and delays in its availability, as a new tobacco smoking cessation aid in the US?

**Findings:**

In a mathematical model, making cytisine available immediately could lead approximately 71 000 more people to quit smoking over 1 year and maintain long-term abstinence, producing more than 500 000 additional life-years. Each additional year of delay in the availability of cytisine might reduce population-level life expectancy by 10 000 years.

**Meaning:**

Cytisine could have major public health benefits, including lengthening population-level life expectancy; therefore, a timely review of cytisine for approval in the US is warranted.

## Teaser

This mathematical model projects the potential population-level health impacts of making cytisine immediately available as an approved smoking cessation aid in the US vs delaying approval by 1 year.

## Introduction

Tobacco smoking is the leading preventable cause of death in the US.^1^ Approximately two-thirds of US adults who smoke report wanting to stop.^2^ No new tobacco cessation medication has been licensed in the US since 2006. Cytisine, a plant-based partial agonist of nicotinic acetylcholine receptors, has been used to treat tobacco dependence for decades.^3^ It is available as a generic or prescription medication in at least 18 countries, was licensed as an over-the-counter natural health product in Canada in 2017, and became available by prescription in the UK in January 2024.^4,5^ In the US, however, it is neither approved for use nor commercially available.

Recent events threaten the timely US regulatory review of cytisine. Cytisine’s US manufacturer planned to submit an application to the US Food and Drug Administration (FDA) for approval of cytisinicline (cytisine’s newer generic name) as a prescription smoking cessation aid in early 2024.^6^ That application included evidence from a US randomized clinical trial reporting 5 times higher odds of tobacco abstinence at 24 weeks for cytisinicline plus behavioral counseling vs placebo plus counseling (odds ratio, 5.3 [95% CI, 2.8-11.1]). The study demonstrated no serious drug-related adverse events.^7^ However, the FDA requested additional data supporting the safety of longer drug administration, delaying the application’s review by at least 1 year.^8^ We estimated the potential life expectancy losses associated with a 1-year delay in the FDA’s consideration of cytisine’s approval.

## Methods

We developed a mathematical model to estimate US life expectancy gains from smoking cessation at different ages (Supplement 1 and Supplement 2). The model accounted for cytisine uptake and effectiveness, noting background levels of smoking cessation and relapse (Table 1).^2,9,10,11,12,13,14^ To capture the outcomes of delayed cytisine approval, we considered immediate treatment initiation and initiation delayed by 1 year. This work followed the Consolidated Health Economic Evaluation Reporting Standards (CHEERS) reporting guideline. It was designated as not human participant research by the Mass General Brigham Human Research Committee. Informed consent was waived per 45 CFR § 46.116.

**Table 1.** Model Input Parameters for Assessing the Health Impact of Immediate vs Delayed Cytisine Availability for Tobacco Cessation

Parameter	Base case value	Range used in sensitivity analysis	References
Prevalence of cigarette smoking in the US in 2021 by age^a^	Age, 18-24 y: 5.3% Age, 25-44 y: 12.6% Age, 45-64 y: 14.9% Age, ≥65 y: 8.3%	NA	Cornelius et al^9^
Background probability of smoking cessation and abstinence for at least 1 y by age^b^	Age, 18-24 y: 9.9% Age, 25-44 y: 8.9% Age, 45-64 y: 5.7% Age, ≥65 y: 5.4%	NA	Babb et al^2^
Relative effectiveness of cytisine: probability of smoking cessation and abstinence for at least 1 y compared with background^c^	2	1.1-2.5	Lindson et al,^10^ assumption^d^
Uptake of cytisine in 1 y among people who smoke	3.8%	1%-10%	Moore et al^11^
Relapse probability after ≥1 y of smoking abstinence	10%	5%-20%	Hughes et al^12^
Life expectancy gain from smoking cessation by age at quit^e^	Quit at age <35 y: 10 y Quit at age 40 y: 9 y Quit at age 50 y: 6 y Quit at age 60 y: 4 y Quit at age 70 y: 1.5 y	NA	Jha et al^13^ and Jia et al^14^

Abbreviation: NA, not applicable.

^a^ Data are shown from the National Health Interview Survey for the civilian, noninstitutionalized population.

^b^ The background probability of smoking cessation is among all people who smoke in a scenario without cytisine available.

^c^ The relative effectiveness of cytisine is applied as a multiplier to the background probability of smoking cessation among people who use cytisine.

^d^ Values were approximations based on the range of effectiveness reported in various studies, including the meta-analysis by Lindson et al.^10^

^e^ Linear interpolation was performed between these quit ages to derive the life expectancy gained from smoking cessation at different ages. For example, people who quit at 45 years of age would gain 7.5 years of life expectancy. We modeled gains in life expectancy for people who quit smoking by 77 years of age; no gain in life expectancy was assumed for people who quit smoking at 78 years or older.^14^

To determine the number of people who smoke and their age distribution, we used 2021 data from the National Health Interview Survey (NHIS) and the Census.^9^ Our analysis included adults aged between 18 and 99 years.

We reviewed historical data about varenicline, a drug with a similar mechanism of action, to determine the number of people who might use cytisine. By 2008, approximately 2 years after FDA approval, varenicline was used by 3.5 million US adults (7.6% of people who smoked).^11,15^ We conservatively halved this proportion to 3.8% to estimate cytisine uptake over 1 year.

We estimated that without cytisine, the age-dependent annual probability of quitting and maintaining abstinence for at least 1 year would be 5.4% to 9.9%; based on data from a meta-analysis, we assumed that cytisine would double this probability (Table 1).^2,10^ We applied a 10% probability of smoking relapse after 1 year of abstinence.^12^

For life expectancy gains from smoking cessation at different ages (compared with continued smoking), we used data from the NHIS, the linked National Death Index, and the Medicare Health Outcome Survey (eMethods in Supplement 1).^13,14^ We assumed that only people who maintain long-term abstinence experience these gains.

To evaluate the impact of delay in availability, we recalculated aggregate life expectancy gains assuming that cytisine-aided smoking cessation occurred 1 year later. For example, a 45-year-old individual who would initiate cytisine if immediately available would instead initiate cytisine at 46 years. The life expectancy gain would be reduced from 7.5 years to 7.2 years, reflecting the later age at quitting.

In sensitivity analysis, we varied cytisine uptake, cytisine’s effectiveness, and relapse probability (Table 1). We evaluated pessimistic and optimistic scenarios, simultaneously setting these parameters to minimize and maximize cytisine’s estimated impact.

## Results

The base case includes an estimated 29.4 million US civilian noninstitutionalized adults who smoke cigarettes (age distribution, 18-24 years: 5.5%; 25-44 years: 37.3%; 45-64 years: 41.8%; ≥65 years: 15.5%). An estimated 1.87 million of these individuals would stop smoking annually and maintain long-term abstinence without cytisine. With a conservative assumption that 3.8% of these individuals would use cytisine in the first year of availability, an estimated 1.12 million people would use the drug in 1 year, and the number of long-term abstainers would increase by 71 000, to 1.94 million. The population-level life-years gained, compared to continued smoking, would increase by 503 000, from 13.23 million (cytisine unavailable) to 13.73 million (cytisine immediately available). If cytisine availability were delayed by 1 year, 10 000 fewer life-years would be gained (Table 2).

**Table 2.** Base Case and Sensitivity Analysis Results for Tobacco Cessation and Life-Year Gains Based on Cytisine Availability^a^

Analysis (base case value; range)	New abstainers^b^	Aggregate life-years gained through smoking cessation	
		Immediate cytisine availability vs status quo^c^	Delayed cytisine availability vs status quo^c^
Base case	71 000	503 000	493 000
Sensitivity to cytisine uptake (3.8%; 1%-10%)^d^	19 000-187 000	132 000-1 323 000	NA
Sensitivity to cytisine effectiveness (2; 1.1-2.5)^e^	7000-107 000	50 000-754 000	NA
Sensitivity to long-term relapse (10%; 20%-5%)^f^	63 000-75 000	447 000-531 000	NA
Pessimistic scenario to optimistic scenario, range^g^	2000-296 000	12 000-2 095 000	NA

Abbreviation: NA, not applicable.

^a^ Results reflect cytisine uptake over 1 year for 2 scenarios, immediate cytisine availability or delayed cytisine availability by 1 year, as well as the downstream health impact. Model results are rounded to the nearest thousand.

^b^ This is the increase in the number of people who would quit smoking and maintain long-term abstinence with cytisine, compared to the status quo scenario in which cytisine is not available but there is background smoking cessation.

^c^ These reflect aggregate life-year gains by the additional people who would stop smoking in scenarios of immediate cytisine availability or delayed cytisine availability compared with the status quo scenario in which cytisine is not available but there is background smoking cessation.

^d^ This is the proportion of all people who smoke who would use cytisine.

^e^ Cytisine effectiveness is measured as a multiplier relative to background smoking cessation probabilities, which reflect both users and nonusers of available pharmacotherapies.

^f^ Relapse is measured as a probability and applied to both the status quo scenario and the scenario in which cytisine is available.

^g^ The pessimistic scenario assumes that 1% of people who smoke use cytisine, the relative effectiveness of cytisine is 1.1, and the relapse probability is 20%. The optimistic scenario assumes that 10% of people who smoke use cytisine, relative effectiveness of cytisine is 2.5, and relapse probability is 5%.

In the sensitivity analysis, cytisine uptake and effectiveness were estimated to have the largest impact on results. Relapse probability, which could affect both status quo and cytisine scenarios, is less influential (Table 2). In the pessimistic or optimistic scenarios, immediate cytisine availability was estimated to produce 2000 or 296 000 additional long-term abstainers and 12 000 or 2.095 million aggregate life-years gained, respectively.

## Discussion

Using a mathematical model, we found that if cytisine were made immediately available in the US and used over 1 year by 3.8% of adults who smoke, more than 500 000 additional life-years were estimated due to increased smoking cessation rates compared with background cessation. Each 1-year delay in cytisine availability could result in 10 000 fewer life-years gained.

New smoking cessation pharmacotherapy options are urgently needed in the US. Utilization of existing FDA-approved pharmacotherapies, such as nicotine replacement products, varenicline, and bupropion, is limited by prior unsuccessful use, concerns about potential adverse effects, supply constraints, and cost. Cytisine may be appealing to many as a naturally derived product, and multiple studies have demonstrated its safety and efficacy.^7,10^

In our base case analysis, we assumed that only approximately 0.5% of US adults who smoke would attain long-term abstinence using cytisine, and only half of these would benefit from cytisine over current cessation practices. Despite these small proportions, both the total number of people who might benefit (71 000) and the potential life-year gains (more than 500 000) are large, the latter because of the major impact of smoking and smoking cessation on life expectancy.^13^ Cessation at older ages still improves life expectancy, but quitting earlier provides greater benefit.^13^

Although long-term drug outcome surveillance is important, we believe that making cytisine available sooner will outweigh the potential disadvantages. First, cytisine has a similar mechanism of action to varenicline, a drug with abundant long-term evidence of safety and efficacy. Indeed, initial reports suggest troubling adverse effects like nausea occur less frequently with cytisine than with varenicline.^5,7,10^ Second, cytisine has been available for many years in other countries, including Canada, without reports of serious cytisine-related adverse effects.^3,16^ Third, the impact of the more common adverse effects associated with cytisine (and varenicline), such as abnormal dreams and insomnia, is likely to be far outweighed by the benefits of earlier smoking cessation.

### Limitations

This study was limited by the uncertainty in the data inputs and assumptions underlying our projections. Potential cytisine uptake is unknown. Nonetheless, if only 1% of US adults who smoke use cytisine over 1 year, instead of 3.8% as assumed in the base case, 132 000 life-years could be gained. Our projections also depend on cytisine’s effectiveness. If cytisine were used only by people who would otherwise use other available pharmacotherapies, its relative effectiveness could be lower than assumed in the base case, where we compared cytisine to background quitting including users and nonusers of available pharmacotherapies. We found that cytisine would still have an important impact on health outcomes even with lower relative effectiveness. Cytisine uptake may also vary across subpopulations of people who smoke, but exactly how remains uncertain. Few published data exist about life expectancy gains from stopping smoking at 65 years or older.^14^

## Conclusions

Even if used by only a small proportion of US adults who smoke tobacco, this mathematical model found that cytisine could have a major impact on public health outcomes. Because quitting sooner is associated with greater life expectancy gains, delayed cytisine availability could decrease health benefits. These findings suggest that a timely FDA review of cytisine is warranted. Delays in the FDA decision could undermine population benefits.

## Notes

Supplement 1. eMethods. Supplemental Information

Supplement 2. Mathematical Model Calculations

Supplement 3. Data Sharing Statement
