# Supplementary material for: Public Health Impact of FDA’s Request for Additional Safety Data on Cytisine for Tobacco Cessation
Source: JAMA Health Forum. 2024 Aug 23;5(8):e242647. doi: 10.1001/jamahealthforum.2024.2647 (PMC11344233; doi:10.1001/jamahealthforum.2024.2647)
Supplement: Supplement 1. — eMethods. Supplemental Information [file jamahealthforum-e242647-s001.pdf]

Supplemental Online Content

Reddy KP, Paltiel AD, Freedberg KA, Rigotti NA. Public health impact of FDA's request for additional safety data on cytisine for tobacco cessation. *JAMA Health Forum.* 2024;5(8):e242647. doi:10.1001/jamahealthforum.2024.2647

eMethods. Supplemental Information

This supplemental material has been provided by the authors to give readers additional information about their work.

© 2024 Reddy KP et al. *JAMA Health Forum.*

SUPPLEMENTARY METHODS

Number of people who would use cytisine

National Health Interview Survey (NHIS) data revealed the following prevalence of cigarette smoking in 2021 among the U.S. civilian, noninstitutionalized population: among people aged 18-24y, 5.3%; among people aged 25-44y, 12.6%; among people aged 45-64y, 14.9%; among people aged ≥65y, 8.3% (prevalence was 11.5% when combining all adult ages).[1] We applied these proportions to the civilian, noninstitutionalized population estimates from the Census to derive the number of U.S. adults who reported current cigarette smoking in 2021, by age.[2]

For the National Health Interview Survey itself, annual surveys in recent years confirm notably lower smoking prevalence among people aged 18-24 years compared with people aged 25-44 years.[3] A different survey, the National Survey on Drug Use and Health, showed in 2019-2020 (for people with "no past-year mental health condition") smoking prevalence of 9.7% among people aged 18-25 years and 16.5% among people aged 26-34 years; the differences by age were even greater among people with mental illness.[4] While the difference in smoking prevalence between 24-year-olds versus 25-year-olds is likely smaller than the difference between the group of 18-24-year-olds versus the group of 25-44-year-olds, more granular data on smoking data by age are not available and would be less precise. Our model estimates are essentially "averages" for people in each of these age groups.

We looked to historical data about varenicline to determine the number of people who might use cytisine. Varenicline was approved by the Food and Drug Administration (FDA) in May 2006, and by February 2008, an estimated 3.5 million U.S. adults used varenicline.[5,6] In 2008, cigarette smoking prevalence among U.S. adults was 20.6% (46.0 million people).[7] Therefore, among U.S. adults who smoked cigarettes in 2008, the estimated proportion who had used varenicline was 3.5 million/46.0

© 2024 Reddy KP et al. *JAMA Health Forum.*

million = 7.6%. We assumed that this reflected varenicline uptake over 2 years. Subsequently, varenicline sales fell after the FDA issued a warning in 2009 based on initial reports of serious neuropsychiatric events (this warning was removed in 2016 after a large randomized controlled trial demonstrated no greater incidence of serious neuropsychiatric events with varenicline compared with bupropion or nicotine replacement therapy[8]). We conservatively halved the 7.6% proportion to apply 3.8% as the uptake of cytisine in a single year. We assumed cytisine uptake would be the same across all ages among people who smoke. Data suggest that younger adults are more likely to make a quit attempt and to successfully quit, but older adults are more likely to use pharmacotherapies when they try to quit.[9]

Smoking cessation and relapse

We assumed that, in the absence of cytisine, there is a "background" annual probability of quitting and maintaining abstinence for at least one year, with or without the use of currently available therapies. Based on NHIS data, these annual probabilities for people who smoke aged 18-24y, 25-44y, 45-64y, and ≥65y are 9.9%, 8.9%, 5.7%, and 5.4%.[9,10] In the presence of cytisine, we assumed that people who do not use cytisine (96.2% of people who smoke, in the base case) have these same annual probabilities of smoking cessation and maintenance of abstinence for at least one year, while those who use cytisine (3.8% of people who smoke, in the base case) have a higher annual probability of smoking cessation and maintenance of abstinence for at least one year.

Relapse after one year of abstinence has been estimated to be 10%.[11] We assumed that this was the relapse probability regardless of whether one had used cytisine to aid in cessation.[12] As such, 90% of people who quit smoking and are abstinent one year later would maintain long-term abstinence for the rest of their lifetime. For example, in the absence of cytisine, 8.0% (8.9% abstinent at one year * 90% able to maintain long-term abstinence after one year) of people aged 25-44y who smoke would be able

to quit and maintain long-term abstinence for the rest of their lifetime. For people who use cytisine in an effort to stop smoking, long-term abstinence would be attained by 16.0% (17.8% * 90%).

Life expectancy gains from cessation

An analysis of data from the NHIS and the linked National Death Index (NDI) indicated the gains in life expectancy from smoking cessation at different ages through age 64y.[13] Compared with people who continue to smoke, these gains are: 10 years for people who quit before age 35y (with life expectancy nearly equivalent to that of people who never smoke), 9 years for people who quit at age 40y, 6 years for people who quit at age 50y, and 4 years for people who quit at age 60y.[13–15] We linearly interpolated between these numbers to derive the life expectancy gain for people who quit at other ages, through age 64y. For example, those who quit at age 37y would gain 9.5 years, those who quit at age 45y would gain 7.5 years, those who quit at age 55y would gain 5 years, and those who quit at age 64y would gain 3.2 years. We made the simplifying assumption that only people who maintained long-term abstinence would experience these gains, and that people who relapsed to smoking after a shorter period of abstinence would not experience any life expectancy gain. In line with published data, we modeled no difference in life expectancy gains across quit ages <35y; as such, people who quit smoking before age 35y have no difference in life expectancy compared with people who never smoke.[13–15]

There are few available data about life expectancy gains for people who quit smoking at ages ≥65y. One study, using data from the Cancer Prevention Study II and applying them to the 1990 US population, estimated adjusted life expectancy gains for people who quit smoking at age 65y compared with people who continue to smoke: 2.0 years for men and 3.7 years for women.[16] A more recent microsimulation-based study, using data from the Medicare Health Outcome Survey Cohort 15 (baseline in 2012, follow-up in 2014), reported a life expectancy gain of 1.5 years for people aged 69y who had recently quit

© 2024 Reddy KP et al. *JAMA Health Forum.*

smoking (less than two years prior) compared with people who continued to smoke.[17] This study also reported significant gains in life expectancy from quitting smoking through age 77y; quitting smoking at age 78y or later did not produce significant gains in life expectancy.[17] Accordingly, we modeled a life expectancy gain of 1.5 years for people who quit smoking at age 70y and no gain for people who quit smoking at ages ≥78y; we linearly interpolated between these values and the 4-year gain in life expectancy from quitting smoking at age 60y estimated from NHIS and NDI data (described in the preceding paragraph) to estimate life expectancy gains from quitting smoking in each one-year age increment from ages 65y through 77y.

Impact of continued unavailability of cytisine

We examined how an extension of one year in the unavailability of cytisine would impact aggregate life-year gains among people who smoke. To determine the number of people who would use cytisine after the one-year delay in availability, we subtracted the number of people who would quit according to background probabilities in the current year and then applied the 3.8% probability of cytisine use among all people who smoke one year later. For people aged 18-33 years, based on our modeling of life expectancy gains from quitting before age 35y, there is no impact on life expectancy from a one-year delay in cytisine availability.[13–15]

Sensitivity analysis

We performed sensitivity analysis around key model parameters. These included: the proportion of people who smoke who would use cytisine in a year (1-10%); the effectiveness of cytisine in promoting smoking cessation, relative to the background probability (1.1-2.5); the probability of relapse after one year of abstinence from smoking (5-20%, regardless of whether one had received cytisine); and

© 2024 Reddy KP et al. *JAMA Health Forum.*

pessimistic or optimistic scenarios in which we simultaneously varied each of the three previous parameters in a manner that would either minimize or maximize the impact of cytisine.

© 2024 Reddy KP et al. *JAMA Health Forum.*

SUPPLEMENTARY REFERENCES

1. Cornelius ME, Loretan CG, Jamal A, et al. Tobacco product use among adults - United States, 2021. *MMWR Morb Mortal Wkly Rep*. 2023;72(18):475-483. doi:10.15585/mmwr.mm7218a1

2. US Census Bureau. National Population by Characteristics: 2020-2022. Census.gov. Accessed April 11, 2024. https://www.census.gov/data/tables/time-series/demo/popest/2020s-national-detail.html

3. American Lung Association. Adult cigarette smoking rate trend by sex, race, and age. Accessed June 6, 2024. https://www.lung.org/research/trends-in-lung-disease/tobacco-trends-brief/data-tables/ad-cig-smoke-rate-sex-race-age

4. Loretan CG, Wang TW, Watson CV, Jamal A. Disparities in current cigarette smoking among US adults with mental health conditions. *Prev Chronic Dis*. 2022;19:E87. doi:10.5888/pcd19.220184

5. Jordan CJ, Xi ZX. Discovery and development of varenicline for smoking cessation. *Expert Opin Drug Discov*. 2018;13(7):671-683. doi:10.1080/17460441.2018.1458090

6. Moore TJ, Cohen MR, Furberg CD. *Strong Safety Signal Seen for New Varenicline Risks*. The Institute for Safe Medication Practices. https://www.ismp.org/sites/default/files/attachments/2018-01/2007Q4.pdf

7. Centers for Disease Control and Prevention (CDC). Cigarette smoking among adults and trends in smoking cessation - United States, 2008. *MMWR Morb Mortal Wkly Rep*. 2009;58(44):1227-1232.

8. Anthenelli RM, Benowitz NL, West R, et al. Neuropsychiatric safety and efficacy of varenicline, bupropion, and nicotine patch in smokers with and without psychiatric disorders (EAGLES): a double-blind, randomised, placebo-controlled clinical trial. *Lancet*. 2016;387(10037):2507-2520. doi:10.1016/S0140-6736(16)30272-0

9. Babb S, Malarcher A, Schauer G, Asman K, Jamal A. Quitting smoking among adults - United States, 2000-2015. *MMWR Morb Mortal Wkly Rep*. 2017;65(52):1457-1464. doi:10.15585/mmwr.mm6552a1

10. Centers for Disease Control and Prevention. Smoking Cessation: Fast Facts. Centers for Disease Control and Prevention. Published August 22, 2022. Accessed April 11, 2024. https://www.cdc.gov/tobacco/data_statistics/fact_sheets/cessation/smoking-cessation-fast-facts/index.html

11. Hughes JR, Peters EN, Naud S. Relapse to smoking after 1 year of abstinence: a meta-analysis. *Addict Behav*. 2008;33(12):1516-1520. doi:10.1016/j.addbeh.2008.05.012

12. Agboola SA, Coleman T, McNeill A, Leonardi-Bee J. Abstinence and relapse among smokers who use varenicline in a quit attempt-a pooled analysis of randomized controlled trials. *Addiction*. 2015;110(7):1182-1193. doi:10.1111/add.12941

13. Jha P, Ramasundarahettige C, Landsman V, et al. 21st-century hazards of smoking and benefits of cessation in the United States. *N Engl J Med*. 2013;368(4):341-350. doi:10.1056/NEJMsa1211128

© 2024 Reddy KP et al. *JAMA Health Forum.*

14. Thomson B, Emberson J, Lacey B, et al. Association between smoking, smoking cessation, and mortality by race, ethnicity, and sex among US adults. *JAMA Netw Open*. 2022;5(10):e2231480. doi:10.1001/jamanetworkopen.2022.31480

15. Thun MJ, Carter BD, Feskanich D, et al. 50-year trends in smoking-related mortality in the United States. *N Engl J Med*. 2013;368(4):351-364. doi:10.1056/NEJMsa1211127

16. Taylor DH, Hasselblad V, Henley SJ, Thun MJ, Sloan FA. Benefits of smoking cessation for longevity. *Am J Public Health*. 2002;92(6):990-996. doi:10.2105/ajph.92.6.990

17. Jia H, Lubetkin E. Use of a microsimulation method for assessing dynamics of smoking status and gains in life expectancy after quitting in a longitudinal cohort of US older adults. *BMJ Open*. 2022;12(11):e062189. doi:10.1136/bmjopen-2022-062189

© 2024 Reddy KP et al. *JAMA Health Forum.*
