# Supplementary material for: Public Health Impact of FDA’s Request for Additional Safety Data on Cytisine for Tobacco Cessation
Source: JAMA Health Forum. 2024 Aug 23;5(8):e242647. doi: 10.1001/jamahealthforum.2024.2647 (PMC11344233; doi:10.1001/jamahealthforum.2024.2647)
Supplement: Supplement 3. — Data Sharing Statement [file jamahealthforum-e242647-s003.pdf]

Data Sharing Statement

Reddy. Public Health Impact of FDA's Request for Additional Safety Data on Cytisine for Tobacco Cessation. *JAMA Health Forum*. Published August 23, 2024.
doi:10.1001/jamahealthforum.2024.2647

Data
Data available: Yes
Data types: Other (please specify)
Additional Information: Spreadsheet of mathematical model
How to access data: Available in supplemental material
When available: With publication

Supporting Documents
Document types: Other (please specify)
Additional Information: Spreadsheet of mathematical model
How to access documents: Available in supplemental material
When available: With publication

Additional Information
Who can access the data: Anyone
Types of analyses: For any purpose
Mechanisms of data availability: With supplemental material
